# Fatty acid profile driven by maternal diet is associated with the composition of human milk microbiota

**DOI:** 10.3389/frmbi.2022.1041752

**Published:** 2022-11-14

**Authors:** Alan J. Marsh, M. Andrea Azcarate-Peril, Mashael R. Aljumaah, Jessica Neville, Maryanne T. Perrin, Lisa L. Dean, Michael D. Wheeler, Ian N. Hines, Roman Pawlak

**Affiliations:** ^1^ Department of Medicine, Division of Gastroenterology and Hepatology, and UNC Microbiome Core, Center for Gastrointestinal Biology and Disease, School of Medicine, University of North Carolina, Chapel Hill, NC, United States; ^2^ Department of Plant and Microbial Biology, North Carolina State University, Raleigh, NC, United States; ^3^ Department of Botany and Microbiology, College of Science, King Saud University, Riyadh, Saudi Arabia; ^4^ Department of Nutrition Science, East Carolina University, Greenville, NC, United States; ^5^ Department of Nutrition, University of North Carolina Greensboro, Greensboro, NC, United States; ^6^ Department of Food, Bioprocessing, and Nutrition Science, North Carolina State University, Raleigh, NC, United States

**Keywords:** human breast milk, microbiota, microbiome, trans fat, diet, nutrition, vegan, vegetarian

## Abstract

Little is known regarding the impact of diet on the breast milk microbiome. We hypothesized that vegan, vegetarian, and omnivore diets would impact the human milk microbiota. We also aimed to explore associations between human milk fatty acid concentrations and microbial composition. A cross-sectional microbiome diversity analysis of human milk samples (N = 72) was performed using 16S rRNA amplicon sequencing. Human milk microbial diversity was not associated with diet type. However, analysis of microbiome in relation to fatty acid profiles revealed significant differences in the overall composition of the human milk microbiota between high (> 0.7% of total fat) and low (< 0.7%) trans-fatty acid groups (TF) (*p* = 0.039, pairwise PERMANOVA *p* = 0.035), high (> 40%) versus low (< 40%) saturated fatty acids (UniFrac *p* = 0.083, PERMANOVA *p* = 0.094), and high (>60%) versus low (<60%) unsaturated fatty acids (UF) (UniFrac *p* = 0.094, PERMANOVA *p* = 0.093). 84% of samples from omnivore mothers were in the high TF group compared to only 12% of samples from vegans. Gut-associated species (*Faecalibacterium*, *Blautia*, *Roseburia* and *Subdoligranulum*) and *Lactobacillus* were characteristic of both high UF and TF groups, but not the low-fat groups. Functional analysis revealed 2,4-dichlorophenol 6-monooxygenase was differentially abundant in the high UF group. Although microbiome diversity did not differ by diet type, TF breast milk content differed by diet group, highlighting the relationship between maternal diet and the microbial profile of human milk.

## Introduction

Human milk is a complex biofluid that supplies nutrients and other compounds such as immunomodulatory components, hormones, and enzymes, which are essential for infant immunological and metabolic health, growth, and development (Ip et al., 2007). In addition, breastfeeding provides early-life protection against gastrointestinal infections and atopic eczema, and promotes cognitive development (Kramer et al., 2001; Kramer et al., 2008).

Breastfeeding also affects the establishment of the infant gut microbiota during the crucial window of microbiome development (Koenig et al., 2011). First, through the provision of non-digestible human milk oligosaccharides, human milk enhances the profile of beneficial bacteria, like bifidobacteria, which can selectively utilize the complex sugar polymers for an ecological advantage (Yu et al., 2013; Lugli et al., 2020). Second, human milk harbors a complex microbiota that allows for vertical transmission of organisms from the mother to the infant’s gastrointestinal (GI) tract (Asnicar et al., 2017; Pannaraj et al., 2017; Korpela et al., 2018). Studies have demonstrated that the intestinal microbiota of breastfed infants differs from that of non-breastfed infants, with the former possessing a higher content of health-promoting bacteria (Bezirtzoglou et al., 2011; Korpela et al., 2018; Ho et al., 2018).

Culture-based analyses first reported a human milk microbiota comprised of *Staphylococcus* and *Streptococcus* (Heikkila and Saris, 2003), but culture-independent analyses have since shown that the human milk microbiota is much more diverse (Lackey et al., 2019). Although studies report substantial inter- and intra-individual variability in microbial composition, most bacterial species in human milk belong to four phyla: *Bacillota*, *Pseudomonadota*, *Actinomycetota*, and *Bacteroidota* (Togo et al., 2019). The genera most frequently identified include *Staphylococcus*, *Streptococcus*, *Lactobacillus*, *Pseudomonas*, *Bifidobacterium*, *Corynebacterium*, *Enterococcus*, *Acinetobacter*, *Rothia*, *Cutibacterium*, *Veillonella*, and *Bacteroides* (Togo et al., 2019; Zimmermann and Curtis, 2020). Exposure to this array of microbes, with culture-based estimates at 10^4^-10^6^ per day (Heikkila and Saris, 2003; Boix-Amoros et al., 2016), is believed to play an important role in seeding the developing infant gut microbiome.

Evidence for the source of organisms in human milk currently supports two theories. First, a retrograde mechanism where the microbiota of the infant oral cavity (via saliva and regurgitation) colonizes the milk ducts. This is supported by evidence showing that pumped milk contains a significantly different microbiota to breast-fed milk, with lower abundance of bifidobacteria and enriched with opportunistic bacteria (Moossavi et al., 2019; Moossavi and Azad, 2020). The second theory is termed “entero-” or “oro-mammary translocation” and hypothesizes that bacteria from the GI tract and oral cavity can travel *via* the immune system to reach the breast (Moossavi and Azad, 2020). Evidence for this route of transmission includes the detection of orally ingested maternal strains in infants’ stools (Jost et al., 2014; Asnicar et al., 2017; Biagi et al., 2017). Furthermore, colostrum and breast tissue harbor microorganisms prior to infant contact, suggesting either a previous external skin colonization, or an internal source (Urbaniak et al., 2014a; Drago et al., 2017).

The influence of diet on the gut microbiota is well-established, as are the differences between high-fiber vegan and vegetarian diets compared to those high in fat (Savage et al., 2018; Wong et al., 2018; Tomova et al., 2019). Research regarding the impact of diet on the human milk microbiota is limited, but recent studies have demonstrated that diet can impact the diversity and composition of the human milk microbiota (Williams et al., 2017; Cortes-Macias et al., 2021a). We and others have shown that maternal diets also impact the fatty acid profile of human milk (Perrin et al., 2019; Tian et al., 2019).

In this study, we hypothesized that maternal vegan, vegetarian, and omnivore diets would have an impact on the microbiota of human milk. The impact of vegan and/or vegetarian diet on milk microbiota has not previously been assessed. In addition, we investigated whether the fatty acid profile of human milk, i.e., saturated, unsaturated, and trans-unsaturated fatty acids, impacted the milk microbiota.

## Materials and methods

### Recruitment and screening

The study protocol was approved by the institutional review board (IRB) at the University of North Carolina Greensboro and East Carolina University. Participants were recruited through vegetarian and vegan organizations, faith-based institutions, and online parenting and breastfeeding support groups. A total of 74 samples were collected of which 72 were included in this study. All methods were carried out in accordance with relevant guidelines and regulations. Informed consent was obtained from all subjects as per the IRB-approved protocol.

An online screening questionnaire (SQ) was used to screen individuals for eligibility. Within the SQ, potential participants were asked about their current diet type (i.e., vegan, vegetarian, omnivore) and breastfeeding status. Once eligibility was determined, individuals were invited to participate. Invited individuals were sent an IRB-approved consent form along with a description of how to collect, store, and ship samples to our lab. In addition, they were asked to complete a diet survey that contained a 15-question food frequency questionnaire (FFQ) along with seven questions related to their use of dietary supplements. Data collected *via* FFQ was used to verify the self-reported diet adherence. Respondents were given a $25 gift card to thank them for participation. Recruitment and sample collection took place from November 2016 to April 2017.

### Criteria of inclusion/exclusion

Inclusion criteria included: United States residency, maternal age 18-46 years old, giving birth to a healthy term infant (i.e., ≥37 weeks’ gestation) who was at least two weeks old at the time of milk sample collection, willing to collect a human milk sample in compliance with the study collection protocol, and willing to complete a diet survey. Criteria of exclusion included: being pregnant, having a methylene tetrahydrofolate reductase gene mutation, inflammatory bowel disease, celiac disease, advanced liver disease (e.g., cirrhosis and hepatitis), hypothyroidism, hyperthyroidism, myeloproliferative disorders, and history of bariatric surgery.

### Diet definition

Vegans were defined as those who did not ingest any meat and who may have ingested animal products no more frequently than less than once per month. Vegetarians were defined as those who did not ingest any meat but regularly ingested other animal products, like eggs and/or dairy.

### Breast milk sample collection

Each participant provided one breast milk sample for analysis. Participants were instructed to collect their breast milk sample in a dimly lit room to preserve light-sensitive nutrients, during the first or second feed of the day, which was at least two hours since the previous feed. Additionally, participants were instructed to express the full content of one breast using the expression method of their choice (i.e., hand expression, breast pump). Next, participants were to transfer the expressed breast milk to a sterile storage bag appropriate for freezing breast milk, label the bag with the collection date, wrap the bag in aluminum foil for protection from the light, and store in the freezer. Samples were either retrieved in-person or shipped on dry ice to the laboratory at the University of North Carolina at Greensboro, where they were thawed, aliquoted, and stored at −20°C until analysis. Frozen samples were delivered on dry ice to the Microbiome Core at the University of North Carolina at Chapel Hill for microbiome analysis, and to North Carolina State University for fatty acid analysis. Methods for fatty analysis have previously been reported (Perrin et al., 2019), and are used for secondary analysis with microbiome data for this study.

### Fatty acid analysis

The fatty acids present in the samples were measured as fatty acid methyl esters according to the method of Bannon et al. (Bannon et al., 1987). Briefly, 0.2 g of milk was hydrolyzed using 1.0 mL of 0.5 N methanolic sodium hydroxide (Thermo Fisher Scientific, Fair Lawn, NJ) for 5 min. The resulting fatty acids were converted to their methyl esters by the addition of 1.0 mL of boron trifluoride (Sigma Chemical Corp., St. Louis, MO) as a catalyst and refluxing for an additional 10 min. The methyl esters were extracted into 1.0 mL of hexane. The resulting fatty acid methyl esters were analyzed using a Perkin Elmer Autosampler XL GC (Perkin Elmer Instruments, Norwalk, CN) and a BPX-70 column (SGE Analytical Science, Austin, TX) with a flame ionization detector (FID) and a capillary column containing 70% cyanopropyl polysilphenylene-siloxane as the stationary phase (30 m length, 0.25 mm i.d., 0.25 mm film thickness). Helium was used as the carrier gas at 1.85 mL/min. A temperature program was used with an initial temperature of 60°C held for 2 min. The temperature was increased to 180°C at 10°C/min, then to a final temperature of 235°C at 4°C/min. The injector was heated to 265°C and the split flow was 76.9 mL/min. The detector temperature was 265°C. Fatty acids were identified by comparison with fatty acid methyl ester standards purchased from Matreya (Matreya, Inc., Pleasant Gap, PA). The amount of each fatty acid present was calculated by normalizing the peak area for each fatty acid identified against the total peak area according to AOCS Method Ce 1b-89 (Firestone, 2009).

### DNA isolation

Samples were transferred into sterile 2 ml microfuge tubes that contained 200 mg of ≤106 μm glass beads (Sigma, St. Louis, MO, USA) and 0.4 ml of ATL buffer (Qiagen, Valencia, CA, USA), supplemented with 60 mg/ml lysozyme (Thermo Fisher Scientific, Grand Island, NY, USA). The suspension was incubated for 10 minutes at 95°C, followed by another round of incubation for 1 hour at 37°C with occasional agitation. Next, the suspension was agitated for 40 minutes on a digital vortex mixer (VWR) at 2,000 rpm and supplemented with 600 international units (IU) of broad-spectrum Proteinase K (Qiagen), 0.3 ml of AL buffer (Qiagen), and incubated overnight at 55°C. The following day, after a brief centrifugation, supernatants were aspirated and transferred into to a new microfuge tube containing 0.3 ml ethanol. DNA was purified using a standard on-column purification method with AW1 and AW2 buffers (Qiagen) as washing agents and eluted in DNase free water. A negative extraction control was included with the run as low-biomass samples can present with extraneous sequencing artifacts due to reagent contaminants (Douglas et al., 2020a).

### 16S rRNA gene amplification and sequencing

Total DNA (12.5 ng) was amplified using universal primers targeting the V4 region of the bacterial 16S rRNA gene as described (Allali et al., 2017; Arnold et al., 2021). Briefly, primers F515/R806 (Caporaso et al., 2012; Kozich et al., 2013) with overhang adapters appended to the 5’ end of each primer for compatibility with Illumina sequencing platform were used. Master mixes contained 12.5 ng of total DNA, 0.5 μm of each primer, and 2X KAPA HiFi HotStart ReadyMix (KAPA Biosystems, Wilmington, MA, USA). Each amplicon was purified using the AMPure XP reagent (Beckman Coulter, Indianapolis, IN, USA). Next, each sample was amplified using a limited cycle PCR program, adding the index adapter sequences (Index 1 (i7) and Index 2 (i5)) and dual‐index barcodes to the amplicon target (Illumina, San Diego, CA, USA). The DNA libraries were then re-purified using the AMPure XP reagent, quantified, and normalized prior to pooling. The DNA library pool was denatured with sodium hydroxide, diluted with Hybridization Buffer (HT1) and heat denatured before being loaded on the MiSeq Reagent Cartridge (Illumina) and the MiSeq System (Illumina). Automated cluster generation and paired-end sequencing with dual reads were performed according to manufacturer instructions.

### Data analysis

Sequencing output from the Illumina MiSeq platform was converted to fastq format and demultiplexed using Illumina Bcl2Fastq 2.18.0.12. The resulting paired-end reads were processed using QIIME 2 2018.11 (Mohsen et al., 2019). Index and linker primer sequences were trimmed using the QIIME 2 invocation of cutadapt. The resulting paired-end reads were processed with DADA2 (Callahan et al., 2016) through QIIME 2 (Bolyen et al., 2019). Reads were trimmed of adapters and reads less than 220 base pairs were eliminated. Reads greater than 220 base pairs were truncated to 220 base pairs and submitted to quality filtering and denoising. Amplicon sequencing units from DADA2 were assigned taxonomic identifiers with respect to SILVA (Quast et al., 2013) using the QIIME 2 q2-featureclassifier. Alpha diversity with respect to: Faith PD whole tree, Evenness (Shannon) index, and observed species number metrics; was estimated using QIIME 2 at a rarefaction depth of 5,000 sequences per subsample. Beta diversity estimates were calculated within QIIME 2 using weighted and unweighted Unifrac distances as well as Bray-Curtis dissimilarity between samples at a subsampling depth of 5,000. Results were summarized, visualized through principal coordinate analysis, and significance was estimated as implemented in QIIME 2. Significance of differential abundance was estimated using ANCOM (Mandal et al., 2015) and ANCOM-BC (Lin and Peddada, 2020) as implemented in QIIME 2. Multivariate statistical tools, including pairwise permutational multivariate analysis of variance (PERMANOVA) models, were used to assess among- and between-group distances and to determine the presence of significant differences. PICRUSt2 (Douglas et al., 2020b) was used to infer function based on 16S community analysis, and significant differences between sample groups were determined using PERMANOVA models. Diversity analysis and visualization were performed using R (version 4.0.3) Phyloseq. Species indicator analysis (SIA) was applied to determine the bacterial taxa most likely responsible for differences between diet/fatty acid groups using the R package (‘indicspecies’) with 9999 permutations and p-value cutoff <0.05 (De Caceres and Legendre, 2009). Taxonomic identity using SIA was frequently only resolved to the genus level. To construct the phylogenetic tree, the ASV table was collapsed at the genus level and trimmed to include one representative sequence for each ASV, with unknowns removed. The filtered set of sequences were aligned, and a phylogenetic tree was generated using Fasttree and visualized using the interactive tree of life (iToL).

## Results

### Sample characteristics

**Figure 1:**
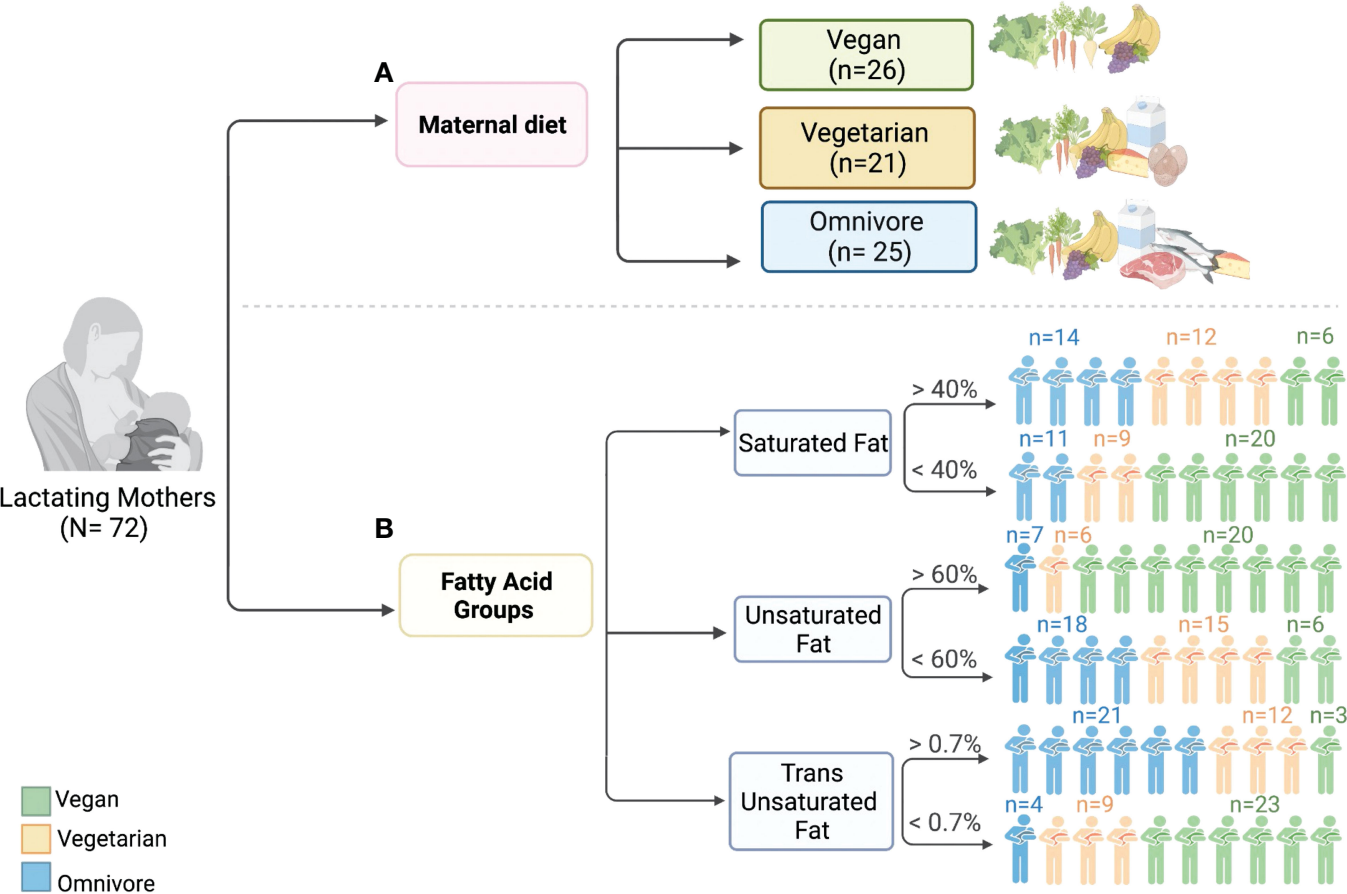
Graphical representation of overall study design including **(A)** diet groups, and **(B)** fatty acid groups.

A total of 72 samples from lactating mothers were analyzed in this cross-sectional study (**Figure 1**; **Table S1**). The mean age of the mothers was 32.1 ± 4.9 years, while infants’ age ranged from 3.5–186 weeks. Mothers had a mean weight of 146 ± 26.2 pounds and a mean BMI of 24 ± 3.8 kg/m^2^. Based on responses to a diet survey, 26 were classified as vegan, 21 as vegetarian, and 25 as omnivore. No significant differences in diversity or overall composition of the microbiome were identified based on BMI (ideal: 18.5-25 kg/m^2^; or high: >25 kg/m^2^), and infant age (three groups: 2-10 weeks, 10-24 weeks, 24-186 weeks) (**Tables 1**, **2**).

**Table 1:** Alpha diversity measuring evenness and phylogenetic diversity within each category.

	Evenness/Distribution							Faith PD						
Categorical	Kruskal-Wallis (all groups)		Kruskal-Wallis (pairwise)					Kruskal-Wallis (all groups)		Kruskal-Wallis (pairwise)				
	H	p-value	Group 1	Group 2	H	p-value	q-value	H	p-value	Group 1	Group 2	H	p-value	q-value
Weight (lbs)	0.004	0.951	gt145 (n=35)	lt145 (n=37)	0.004	0.951	0.951	0.126	0.723	gt145 (n=35)	lt145 (n=37)	0.126	0.723	0.723
BMI	0.599	0.439	High (n=26)	Ideal (n=46)	0.599	0.439	0.439	0.045	0.833	High (n=26)	Ideal (n=46)	0.045	0.833	0.833
Infant Age (weeks)	0.852	0.653	1 (n=7)	2 (n=27)	0.436	0.509	0.585	1.285	0.526	1 (n=7)	2 (n=27)	1.473	0.225	0.613
			1 (n=7)	3 (n=38)	0.613	0.434	0.585			1 (n=7)	3 (n=38)	0.565	0.452	0.613
			2 (n=27)	3 (n=38)	0.298	0.585	0.585			2 (n=27)	3 (n=38)	0.256	0.613	0.613
Saturated Fat	3.844	0.050	gt40 (n=32)	lt40 (n=40)	3.844	0.050	0.050	0.647	0.421	gt40 (n=32)	lt40 (n=40)	0.647	0.421	0.421
Unsaturated Fat	4.208	0.040	gt60 (n=33)	lt60 (n=39)	4.208	0.040	0.040	0.023	0.879	gt60 (n=33)	lt60 (n=39)	0.023	0.879	0.879
Trans Fats	0.107	0.744	gt0.7 (n=36)	lt0.7 (n=36)	0.107	0.744	0.744	0.772	0.380	gt0.7 (n=36)	lt0.7 (n=36)	0.772	0.380	0.380
Diet Group	1.585	0.453	Omnivore (n=25)	Vegetarian (n=21)	1.392	0.238	0.564	2.826	0.243	Group1 (n=25)	Group2 (n=21)	1.839	0.175	0.263
			Omnivore (n=25)	Vegan (n=26)	0.784	0.376	0.564			Group1 (n=25)	Group3 (n=26)	0.157	0.692	0.692
			Vegetarian (n=21)	Vegan (n=26)	0.202	0.653	0.653			Group2 (n=21)	Group3 (n=26)	2.440	0.118	0.263

**Table 2:** Beta diversity values comparing groups within each category.

	Bray Curtis				Unweighted				Weighted			
	PERMANOVA		PARIWISE PERMANOVA		PERMANOVA		PARIWISE PERMANOVA		PERMANOVA		PARIWISE PERMANOVA	
**Parameter**	Pseudo-F	p-value	p-value	q-value	Pseudo-F	p-value	*P* value	q-value	Pseudo-F	p-value	*P* value	q-value
**BMI**	0.821422	0.806	0.819	0.819	0.82231	0.959	0.957	0.957	1.07156	0.351	0.339	0.339
**Saturated Fat**	1.16181	0.188	0.205	0.205	1.00509	0.408	0.436	0.436	1.62553	0.084	0.094	0.094
**Trans Fat**	1.06323	0.335	0.349	0.349	0.984109	0.483	0.487	0.487	1.98651	0.039	0.035	0.035
**Unsaturated Fat**	1.0713	0.324	0.312	0.312	1.00002	0.43	0.449	0.449	1.56506	0.094	0.093	0.093
**Weight lbs**	0.893621	0.682	0.629	0.629	0.876529	0.853	0.832	0.832	1.11627	0.335	0.284	0.284

### The human milk microbiome

After filtering for quality, a total of 5,738,640 non-chimeric reads corresponding to 79,703 reads per sample were found. Over 99% of the filtered reads were assigned to a taxonomic group, while the remaining reads were unassigned.

An average of 10 phyla were detected per human milk sample, with 28 bacterial phyla identified in total (**Figure S1**). Of these, three phyla were identified in all samples (*n*=72): *Actinomycetota, Bacillota*, and *Pseudomonadota*, which together comprised over 85% of reads in most samples (*n*=68). *Bacillota* had the highest relative abundance with an average of 43.5 ± 19.2%, followed by *Pseudomonadota* (41.7 ± 20.3%) and *Actinomycetota* (8.8 ± 6.4%). *Bacteroidota* were present in 70 samples but at a lower relative abundance (2.63 ± 3.8%).

An average of 83 ± 37.8 genera were assigned to each sample. *Pseudomonas*, *Anoxybacillus* and *Cutibacterium* were present in every sample (*n*=72), while *Lactobacillus* and *Escherichia*-*Shigella* were detected in 71 samples. *Staphylococcus*, *Sphingobium*, *Ralstonia*, *Halomonas* and *Acinetobacter* were each present in 70 samples, and *Streptococcus* in 69 (**Figure S1**). On average, *Anoxybacillus* was the most abundant genus across all samples, representing a mean of 11.46 ± 6.98 percentage of reads, followed by *Lactobacillus* (8.88 ± 9.48), *Pseudomonas* (8.49 ± 10.55) and *Streptococcus* (5.68 ± 14.4). Other notable genera found in the samples included *Ralstonia* (3.11%), *Staphylococcus* (2.13%), *Bacillus* (1.66%), *Anoxybacillus* (1.42%), *Acinetobacter* (1.28%), *Lactobacillus* (1.05%), *Roseburia* (0.24%) and *Cutibacterium* (0.14%). B*ifidobacterium* was detected in 25 samples but never above 1% abundance.

Reads were assigned to a total of 659 genera, but 34.7% of these were not identified in more than one sample, and 77.8% were found in less than ten samples. The negative extraction control resulted in only 2,959 reads where the most abundant phylum was *Pseudomonadota* with 80.84% of the assigned reads, followed by *Bacillota* (10%), *Actinomycetota* (8.18%), and *Bacteroidota* and the *Candidatus* phylum *Dependentiae* (TM6) (both <1%). Reads were assigned to 23 genera. *Pseudomonas* was the most abundant genus with 45.42% of the reads, followed by *Escherichia*-*Shigella* (15.31%).

### Human milk microbiota based on diet group

No statistically significant differences were identified in samples from vegan, vegetarian, and omnivorous mothers in relation to the distribution of species or phylogenetic diversity (**Table S2**; **Figure S2**). Likewise, groups were not significantly different in terms of beta diversity after correction for false discovery rate (FDR).

**Figure 2:**
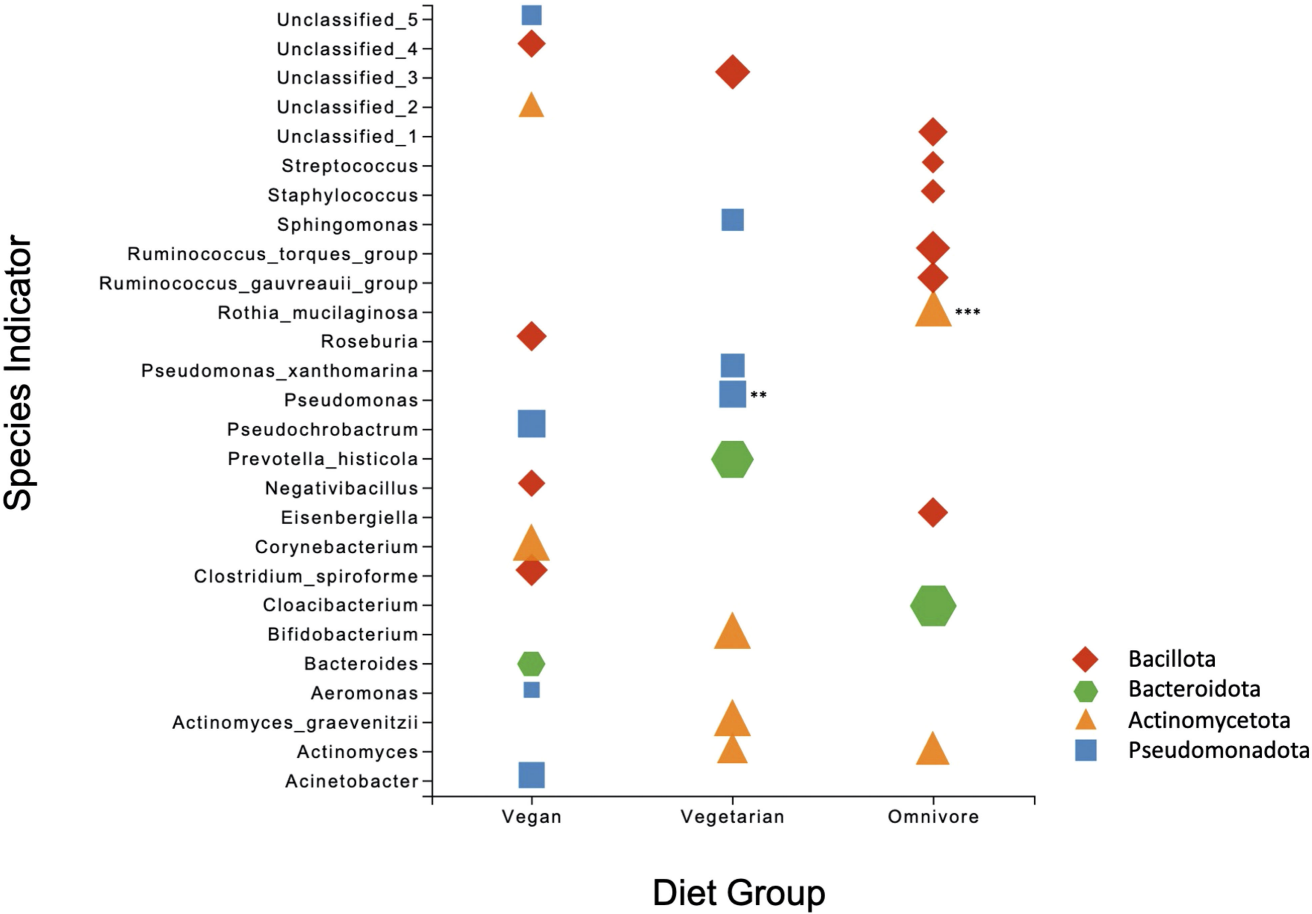
Scatter plot of the Species Indicator Analysis depicting species characteristic of vegan, vegetarian, and omnivore diet groups. All results are significant (*p* = <0.05), with ** signifying *p* = <0.01 and *** *p* = < 0.001. Plot points are color-coded according to phylum, with the size of each point corresponding to specificity (i.e. mean abundance of the ASV in the target group divided by sum of its mean abundance across all groups).

The non-parametric Mann-Whitney U-test with 1% FDR was applied to compare the relative abundances of individual phyla and genera between groups. No significant differences were observed between the diet groups at the phylum level. At the genus level, statistically significant differences were observed between the vegan and omnivore groups (**Table S3**). Of the 18 genera, *Mycobacterium*, *Rothia*, *Geobacillus*, *Actinomyces*, and a genus of the family Vermiphilaceae were present in more than 30 samples and differences in their relative abundance was significant at FDR corrected *p* value < 0.001. The remaining genera were present in low abundance and in less than ten human milk samples. Species indicator analysis (SIA) revealed several species to be significant positive indicators of omnivore (*n*=9), vegetarian (*n*=9), and vegan (*n*=12) diets (**Figure 2**). Of these, *Rothia mucilaginosa* was the most significant indicator (*p* < 0.001), associated with the omnivore group.

### Fatty acid content and microbiota

**Figure 3:**
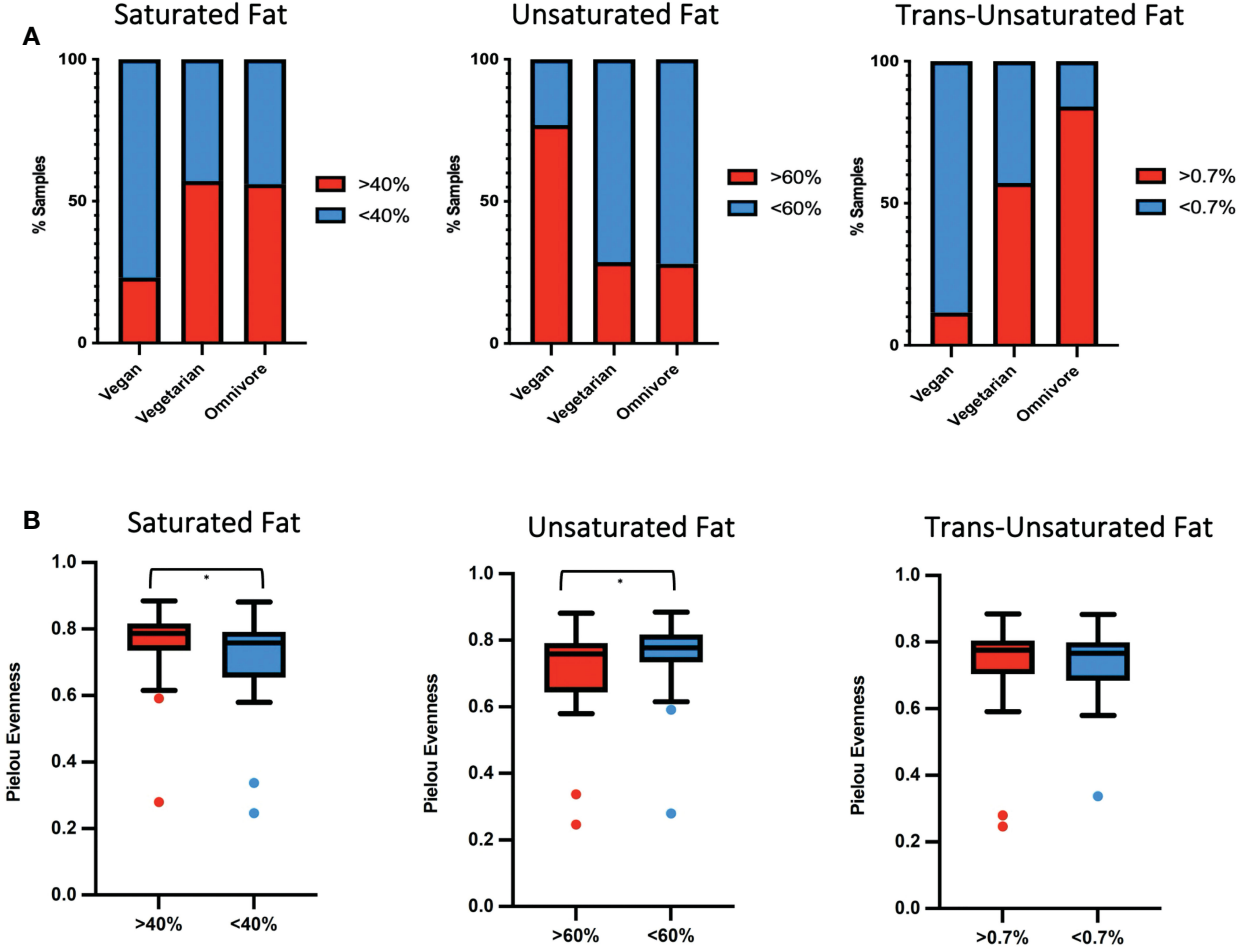
**(A)** Percentage of human milk samples by diet group belonging to either high (red) or low (blue) percentage of fats for SF, UF and TF. **(B)** Box plot showing differences and significance between high and low-fat groups, with * signifying *p* = <0.05.

A previous report by our group showed that among samples included in the present study, the median unsaturated fatty acids as a percentage of total fatty acids was higher in samples from vegan women. The same samples had a lower percentage of saturated and trans-unsaturated fatty acids (Perrin et al., 2019). Based on the fat percent abundance in each sample, we categorized them into low and high groups: saturated fats (SF, lower/greater than 40%), unsaturated fats (UF, lower/greater than 60%), and trans-unsaturated fatty acids (TF, lower/greater than 0.7%). Based on the previous classification, 77% of vegans fell into the high UF category, correlating with plant-based diets being rich in unsaturated fats, while only 12% corresponded to the high TF group (**Figure 3A**). In contrast, 28% of omnivores were categorized in the high UF category, while 84% were high in TF. 29% of vegetarians were in the high UF group and 57% in the high TF group. 56% of omnivores, 57% of vegetarians, and 23% of vegans were categorized in the high SF group.

**Figure 4:**
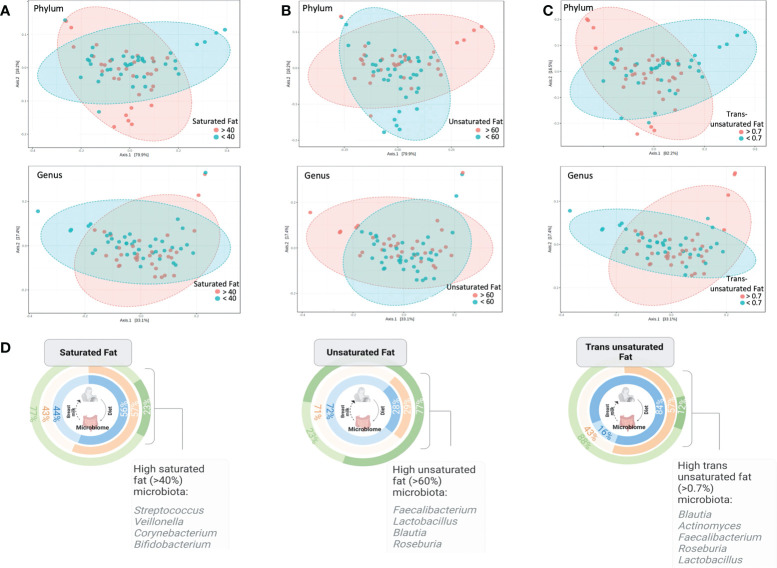
PCoA plots at phylum and genus level based on weighted Unifrac distances between **(A)** high and low SF groups, **(B)** high and low UF groups and **(C)** high and low TF groups (the only significant comparison; PERMANOVA, *p* < 0.05; PERMDISP, *p* = 0.227). **(D)** Illustration showing the percentage of human milk samples belonging to either the high or low SF, UF, or TF fatty acid groups, and color coded according to diet group, along with notable taxa associated with high fat groups as identified by SIA.

In the SF and UF groups, the evenness (Pielou) of the microbiota was found to differ significantly (*p* < 0.05) between high and low groups (**Figure 3B**), while no significant differences were identified in the TF group (**Table S2**). Significant differences in the overall composition of the human milk microbiota were identified between the low and high groups within the TF group (*p* = 0.039, pairwise PERMANOVA *p* = 0.035) (**Figure 4C**; **Table S2**). Additionally, we observed differences in weighted UniFrac-based diversity between high and low groups within both SF (*p* = 0.083, PERMANOVA *p* = 0.094) and UF (*p* = 0.094, PERMANOVA *p* = 0.093) groups (**Figures 4A, B**; **Table S4**).

**Figure 5:**
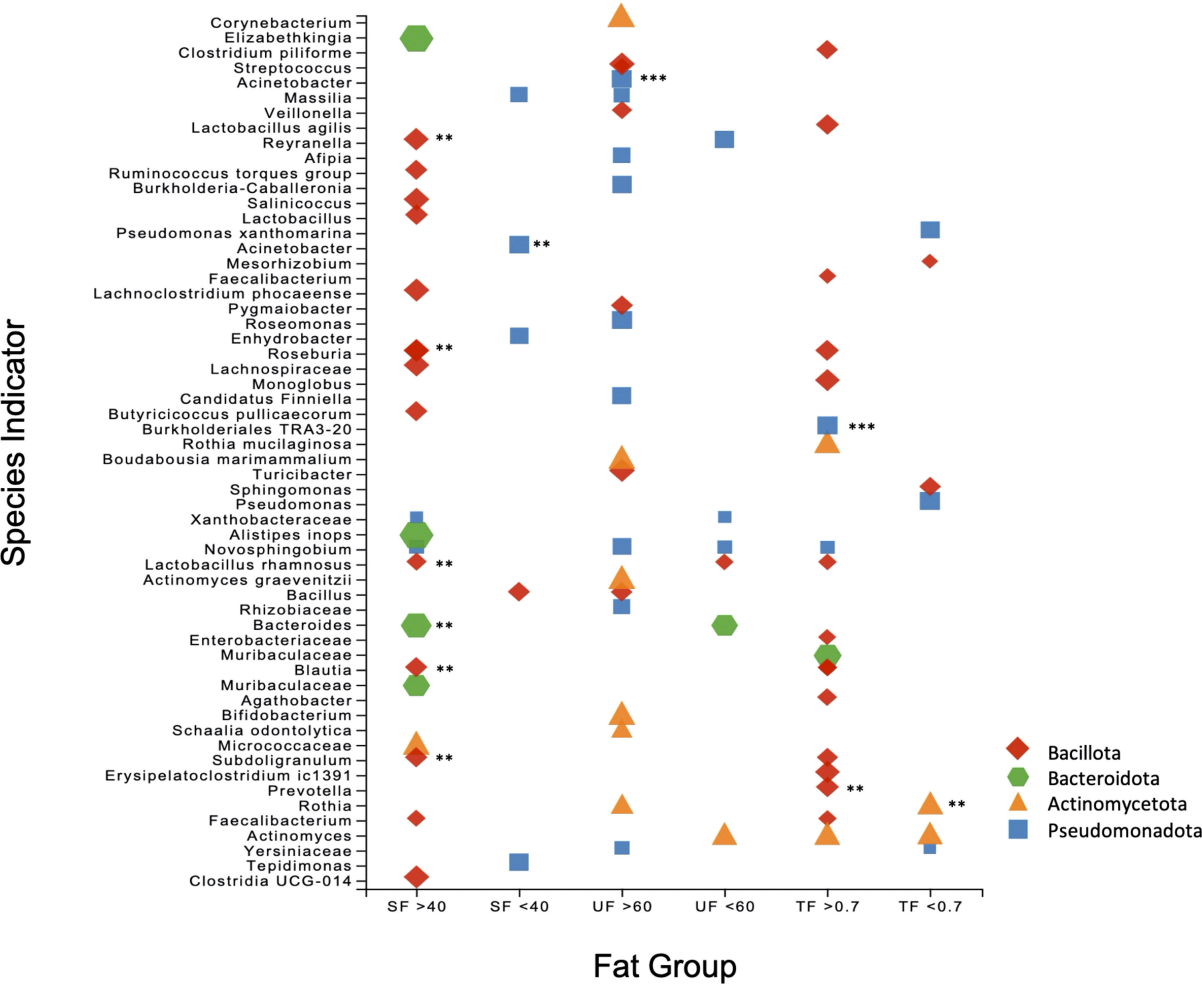
Scatter plot of the Species Indicator Analysis (SIA) depicting species characteristic of high and low groups for SF, UF, and TF. Plot points are color-coded according to phylum, with the size of each point corresponding to specificity (i.e. mean abundance of the ASV in the target group divided by sum of its mean abundance across all groups). All results are significant (*p* = <0.05), with ** signifying *p* = <0.01 and *** *p* = < 0.001.

SIA identified 20 taxa as representative of the high UF group (**Figures 4D**, **5**) including *Lactobacillus rhamnosus* and undetermined species of *Lactobacillus*, 21 taxa as representative of the high SF, including *Streptococcus* and *Bifidobacterium*, and 19 taxa as representative of the TF group, including *L. rhamnosus* and *Lactobacillus agilis*. Of the total number of species, 60.9% and 73.7% identified as characteristic of the high UF and TF groups belonged to the *Bacillota*, respectively. This contrasts with the high SF group which had less representatives of the *Bacillota* (28.6%) and greater proportions of *Pseudomonadota* (42.9%) and *Actinomycetota* (28.6%).

### Metabolic functions associated with different fatty acids

Finally, phylogenetic investigation of communities by reconstruction of unobserved states (PICRUSt) was used to determine differences in predicted KEGG orthology (KO), enzyme classification (EC), and functional pathways for diet groups (vegan, vegetarian, and omnivore) and each of the fat groups (SF, UF, TF). Between the diet groups, no KO functions, enzymes, or pathways were significant by ANCOM differential abundance, or Bray-Curtis pairwise PERMANOVA (**Table S4**).

No KO functions, enzymes, or pathways were differentially abundant between the high and low groups of SF or TF (**Table S3**). Differential abundance analysis by ANCOM-BC of KO and EC within UF revealed a large number of significant hits (6449 and 1975, respectively) in the high compared to the low groups. The top result in both analyses was a 2,4-dichlorophenol 6-monooxygenase (K10676, W = 4.14; EC:1.14.13.20, W = 4.177). Pairwise PERMANOVA showed a significant difference among the high and low TF groups across KO function (q = 0.016), EC (q = 0.02), and pathways (q = 0.016) (**Table S3**). KO function was also significant between high and low SF groups (q = 0.049).

## Discussion

In this study, we analyzed the microbiome of human milk samples from vegan, vegetarian, and omnivore mothers. Additionally, using data profiling the fatty acid composition of all human milk samples (Perrin et al., 2019), we assessed whether high or low saturated fat (SF), unsaturated fat (UF), and trans-unsaturated fats (TF) had an impact on the microbiota of the milk. Although diet groups did not result in significant differences, fatty acids were significantly associated with the human milk microbiota. We demonstrated that human milk containing a greater abundance of TF also exhibit a significantly different human milk microbiota compared to samples that are low in TF.

The genera identified in this analysis are considered typical of the human milk microbiota (Togo et al., 2019; Zimmermann and Curtis, 2020). However, *Anoxybacillus* was the dominant genus across our samples, and while it has been found previously in human milk (Urbaniak et al., 2014b; Urbaniak et al., 2016) and also in the infant gut microbiome (Duan et al., 2018), it is possible that it arose from environmental sample exposure given the thermophilic and spore-forming nature of the genus. Emerging evidence in mice suggests it may correlate negatively with weight gain (Zhang et al., 2019; Huang et al., 2021). Members of the Bacillota phylum are known to harbor multiple and heterogenous copies of the 16S rRNA gene (Perfumo and Marchant, 2010; Sun et al., 2013), which may have resulted in the overestimation of this genus in our data. Although it was not possible with the present study, future studies should aim to recover *Anoxybacillus* isolates from breast milk samples and determine the temperature growth range for this genus, comparing it to other mesophilic *Anoxybacillus* isolates, which is rare for the genus (Paul et al., 2012; Goh et al., 2013).

*Bifidobacterium*, acknowledged for its importance in infant health, was present at a low abundance (<1%), but this finding is common among human milk studies (Zimmermann and Curtis, 2020). Human milk samples in this study were collected without skin sterilization in order to capture the microbiota under natural feeding conditions (Sakwinska et al., 2016). Genera like *Burkholderia*, *Corynebacterium*, *Novosphingobium*, *Propionibacterium*, *Pseudomonas*, *Ralstonia*, *Sphingopyxis*, *Sphingobium*, and *Sphingomonas*, all identified in our samples, have been detected as reagent contaminants (Douglas et al., 2020a), (Salter et al., 2014; Kim et al., 2017; Perez-Munoz et al., 2017), but of these, only *Pseudomonas* and *Ralstonia* were found in our negative control indicating limited reagent contamination.

Overall, differences in diversity between the vegan, vegetarian and omnivore groups were not significant. In contrast, a recent study reported that the human milk microbiota was shaped by maternal dietary clusters that broadly resembled a vegetarian/vegan diet (high intake of plant protein, fiber, and carbohydrates), or an omnivore diet (high intake of animal protein and lipids) (Cortes-Macias et al., 2021a). There are several possible reasons why we did not observe significant differences amongst our samples. Human milk samples in this study represented a wide range of the postpartum period (2 weeks to over 2 years). It is known that the stage of lactation (i.e. colostrum, transitional, or mature milk) impacts the human milk microbiota (Khodayar-Pardo et al., 2014), and while longitudinal studies characterizing human milk throughout the mature stage are lacking, one study demonstrated that *Sphingobium* and *Pseudomonas* became enriched later in lactation (Gonzalez et al., 2021). It is therefore conceivable that this variation may have impacted our ability to make clear distinctions in microbiota. The lack of significance across other metrics (BMI, stage of lactation, diet groups) which have shown significance elsewhere (Khodayar-Pardo et al., 2014; Cortes-Macias et al., 2021a; Cortes-Macias et al., 2021b) may indicate that increasing the power of the study by analyzing a larger number of samples would result in more significant differences between samples.

SIA revealed differences at the species level between samples from women following different diet patterns. Species of *Bacteroides*, *Corynebacterium*, *Acinetobacter*, and *Roseburia* were associated with vegan diets, whereas *Rothia mucilaginoasa* and species of *Staphylococcus*, *Streptococcus* and *Ruminococcus* were characteristic of the omnivore group. In contrast with our study, Cortes-Macias et al. found increased proportions of *Staphylococcus*, *Veillonella*, *Lactobacillus*, and *Bifidobacterium* in their fiber cluster, while *Bacteroides*, *Escherichia*/*Shigella*, and *Gemella* were increased in animal protein and lipids cluster (Cortes-Macias et al., 2021a).

Previous analysis of these samples showed that the different diet groups correlated with differences in the fatty acid profiles of human milk (Perrin et al., 2019), with vegan mothers having a higher percentage of UF but lower TF compared to samples from omnivores, which contained a greater proportion of SF and TF. We identified significant differences in evenness between high and low groups of SF and UF, and most significantly, differences between the bacterial communities of samples containing high and low levels of TF. Our results are in accordance with a previous study linking changes in the human milk microbiota to differences in human milk fat composition (Kumar et al., 2016). SF and mono-UF had been associated with microbiota changes previously, with both groups showing an inverse correlation with *Corynebacterium* (Williams et al., 2017). Species of *Lactobacillus* were positively associated with high TF and high UF groups, supporting a previous finding by Kumar et al. (Kumar et al., 2016).

A range of gut-associated bacteria, including *Faecalibacterium*, *Ruminococcus*, *Prevotella*, *Blautia*, *Veillonella*, and *Bacteroides*, were detected across the samples. This corresponds with observations made in a recent systematic review that showed that 49% of human milk taxa are shared with the gut (Togo et al., 2019). The presence of *Alistipes*, *Bacteroides*, *Blautia*, *Faecalibacterium*, *Prevotella*, and *Roseburia* in both the high UF and TF groups is interesting given that diet is known to be a powerful modulator of the gut microbiome, with diet-influenced, gut-associated genera potentially substantiating the theory of oro-mammary translocation of species from the GI tract to breast ducts (Moossavi and Azad, 2020). The presence of a microbiota characteristic of a specific fat profile may be correlative rather than causative, as mechanistic studies demonstrating the ability of bacterial species to metabolize different fat types are lacking.

Finally, a 2,4-dichlorophenol 6-monooxygenase, involved in pathway for the degradation of aromatic compounds, was differentially abundant in the higher UF group. 2,4-dichlorophenol 6-monooxygenase is an oxidoreductase involved in chlorocyclohexane and chlorobenzene degradation, compounds used in deodorants and pesticides. Environmental contamination of human milk with these chlorinated hydrocarbons has been a concern for many years (Dyment et al., 1971), and can potentially have a negative impact on infant development (Pajewska-Szmyt et al., 2019). The lipophilic nature of chlorinated hydrocarbons allows them to accumulate in human adipose tissue and human milk (Teufel et al., 1990; Malik et al., 2022). Although the human milk samples were not tested for contaminants, except for glycophase (Wooten A et al., 2019), the presence of this oxidoreductase may indicate that increased proportions of UF could facilitate the accumulation of chlorinated hydrocarbons, which is then available for utilization by select bacteria possessing genes for 2,4-dichlorophenol 6-monooxygenase.

The mechanism through which diet shapes the milk microbiota has yet to be determined. Our analysis builds on previous work substantiating that diet impacts the fatty acid composition of human milk (Yahvah et al., 2015) to show that the fatty acid profile of human milk associates with changes in the microbiota, indicating that the relationship between diet and milk microbiota is likely influenced by individual dietary components. Specifically, human milk samples with a higher proportion of TF possessed a significantly different microbiome characterized by species of *Prevotella*, *Burkholderiales*, *Blautia*, *Faecalibacterium*, and *Lactobacillus* compared to the samples with lower trans-unsaturated fatty acids. Furthermore, while the specific diet types did not significantly impact the human milk microbiota, they did correlate closely with the fatty acid profile, again indicating that increased sample size might have resulted in finding significance between different diets. While this study highlights the relationship between maternal nutrition, human milk composition, and the human milk microbiota, the implications of microbial changes to human milk for infant health requires further research.

## Data availability statement

Raw data used in this study are available on the Sequence Read Archive (SRA) under submission number PRJNA848748.

## Ethics statement

The study protocol was approved by the institutional review board (IRB) at the University of North Carolina Greensboro and East Carolina University. The patients/participants provided their written informed consent to participate in this study.

## Author contributions

The authors’ responsibilities were as follows: RP: designed research, conducted research, and aided with manuscript preparation. JN: designed research, conducted the research. MP: designed research, conducted the research. IH: conducted the research. MW: conducted the research. AM, and MA: analyzed the data and performed statistical analyses. AM and M.AA-P had primary responsibility for final content. All authors read and approved the final manuscript. Authors had no conflict of interest.

## Funding

This research received funding from the Vegetarian Nutrition Dietary Practice Group at the Academy of Nutrition and Dietetics, and the East Carolina University College of Allied Health Sciences. The UNC Microbiome Core is supported in part by P30 DK034987 Centre for Gastrointestinal Biology and Disease (CGIBD) and P30 DK056350 UNC Nutrition Obesity Research Center (NORC).

## Conflict of interest

The authors declare that the research was conducted in the absence of any commercial or financial relationships that could be construed as a potential conflict of interest.

## Publisher’s note

All claims expressed in this article are solely those of the authors and do not necessarily represent those of their affiliated organizations, or those of the publisher, the editors and the reviewers. Any product that may be evaluated in this article, or claim that may be made by its manufacturer, is not guaranteed or endorsed by the publisher.

## Abbreviations

SF, saturated fatty acid; UF, unsaturated fatty acid; TF, trans-unsaturated fatty acid; SIA, species indicator analysis; GI, gastrointestinal; IRB, Institutional Review Board; SQ, screening questionnaire; PERMANOVA, pairwise permutational multivariate analysis of variance; FDR, false discovery rate; predicted KO, KEGG orthology.
