# Corrigendum: Fatty acid profile driven by maternal diet is associated with the composition of human milk microbiota

**DOI:** 10.3389/frmbi.2023.1143303

**Published:** 2023-03-03

**Authors:** Alan Marsh, M. Andrea Azcarate-Peril, Mashael Aljumaah, Jessica Neville, Maryanne T. Perrin, Lisa L. Dean, Michael D. Wheeler, Ian N. Hines, Roman Pawlak

**Affiliations:** ^1^ Department of Medicine, Division of Gastroenterology and Hepatology, and UNC Microbiome Core, Center for Gastrointestinal Biology and Disease, School of Medicine, University of North Carolina, Chapel Hill, NC, United States; ^2^ Department of Plant and Microbial Biology, North Carolina State University, Raleigh, NC, United States; ^3^ Department of Botany and Microbiology, College of Science, King Saud University, Riyadh, Saudi Arabia; ^4^ Department of Nutrition Science, East Carolina University, Greenville, NC, United States; ^5^ Department of Nutrition, University of North Carolina Greensboro, Greensboro, NC, United States; ^6^ Department of Food, Bioprocessing, and Nutrition Science, North Carolina State University, Raleigh, NC, United States

**Keywords:** human breast milk, microbiota, microbiome, trans fat, diet, nutrition, vegan, vegetarian

In the published article, there was an error in the section Author Affiliations. Authors Michael D. Wheeler and Ian N. Hines should have affiliation 4, not affiliation 2.

The correct affiliations can be seen below.

Michael D. Wheeler^4^ and Ian N. Hines^4^

*^4^ Department of Nutrition Science, East Carolina University, Greenville, NC, USA*

The authors apologize for this error and state that this does not change the scientific conclusions of the article in any way. The original article has been updated.

