# Supplementary material for: Fatty acid profile driven by maternal diet is associated with the composition of human milk microbiota
Source: Front Microbiomes. 2022 Nov 14;1:1041752. doi: 10.3389/frmbi.2022.1041752 (PMC12993459; doi:10.3389/frmbi.2022.1041752)
Supplement: Supplementary file 1 [file DataSheet_1.pdf]

Supplemental Information:

The mean number of reads per sample after adapter trimming was: 109996.75. The mean number of reads per sample after quality filtering was: 83664.6666.

Figure S1 A) Bar plots showing the different alpha diversity indices for the three diet groups. B) PCA plot illustrating differences among the three diet groups.

Figure S2 A) Phylogenetic tree illustrating the distribution of taxa at the phylum and genus level for OTUs present at >5% abundance across the 16S rRNA dataset. Each genus shown is present in all samples. B) Relative abundance of the most abundant bacterial genera present across the three diet groups: Omnivore (n=25), Vegetarian (n=21), and Vegan (n=26).

Table S1 Characteristics of the mothers and infants included in each diet group, including means and standard deviations.

Table S2 List of genera that were statistically different between omnivores and vegans.

Table S3 Beta diversity values for the PICRUSt functional analyses of the high and low groups for each of the fats (SF, UF, and TF).

Table S4 Beta diversity values for the PICRUSt functional analyses of the diet groups (Vegan, Vegetarian, and Omnivore)

Table S5 Weighted NSTI values for EC and KO analysis for each sample.

Supplemental Figure 1

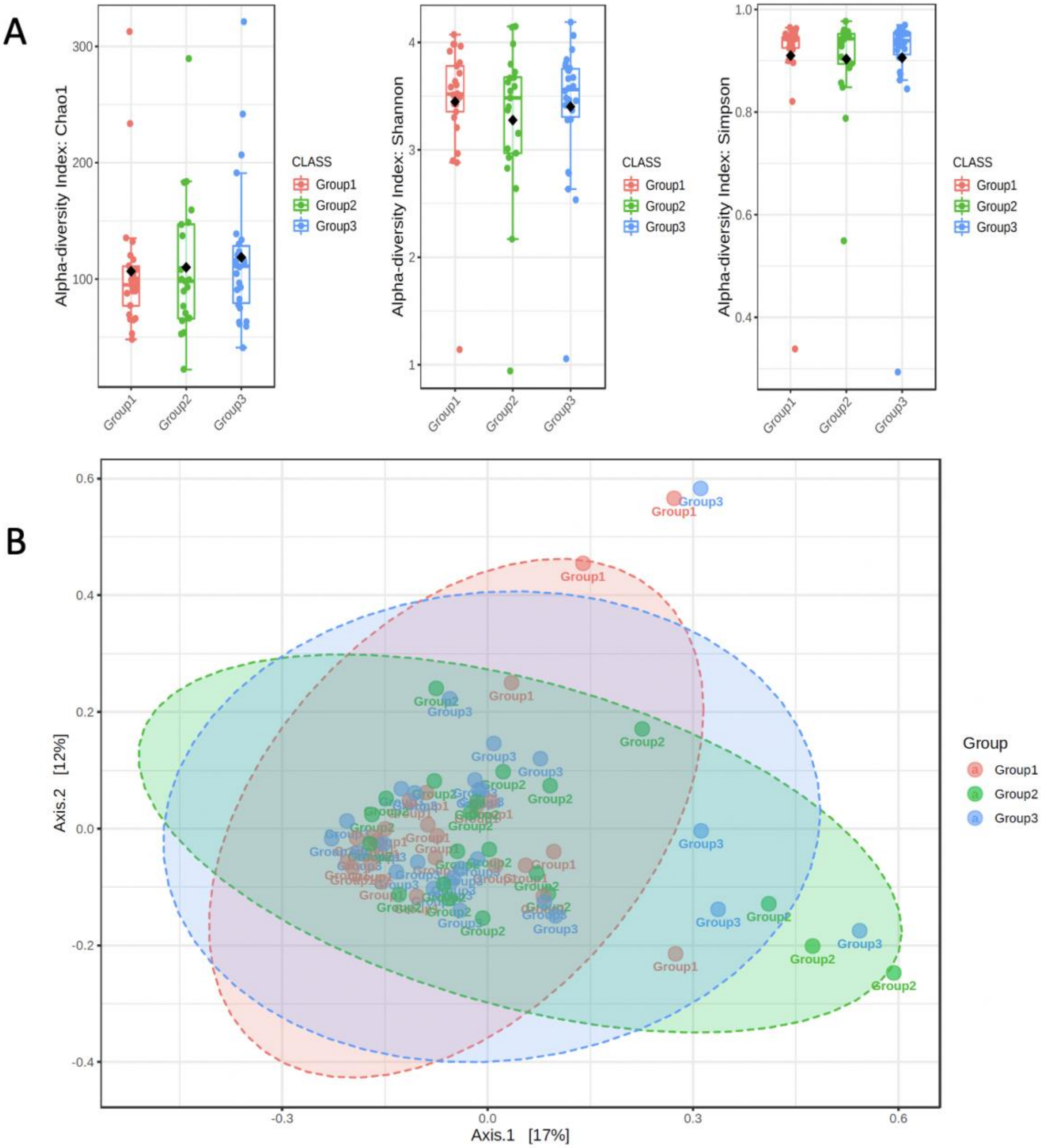

Supplemental Figure 2

A

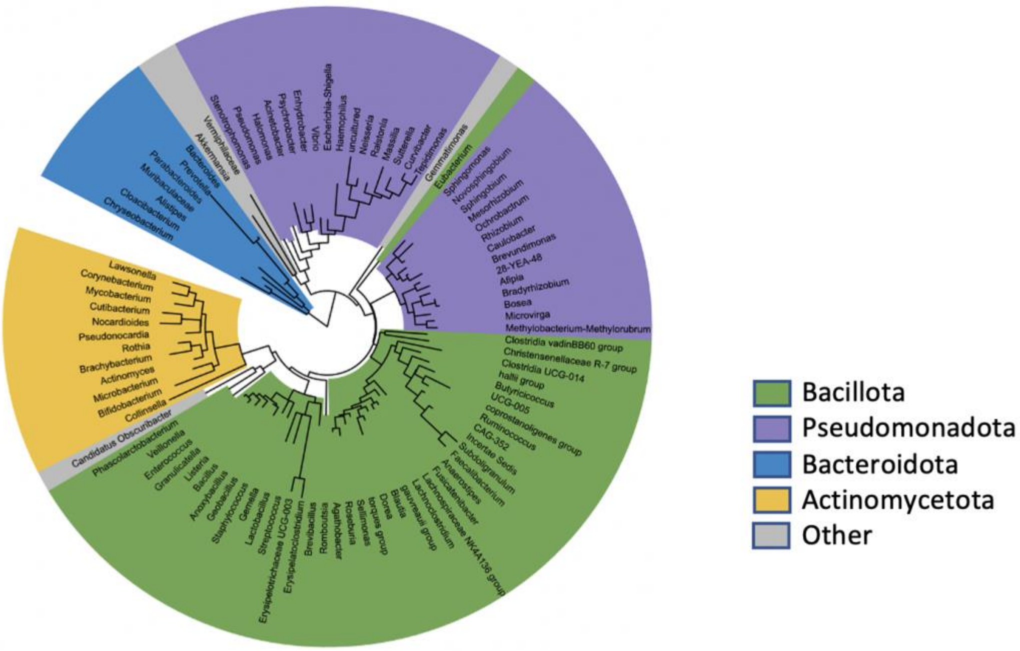

B

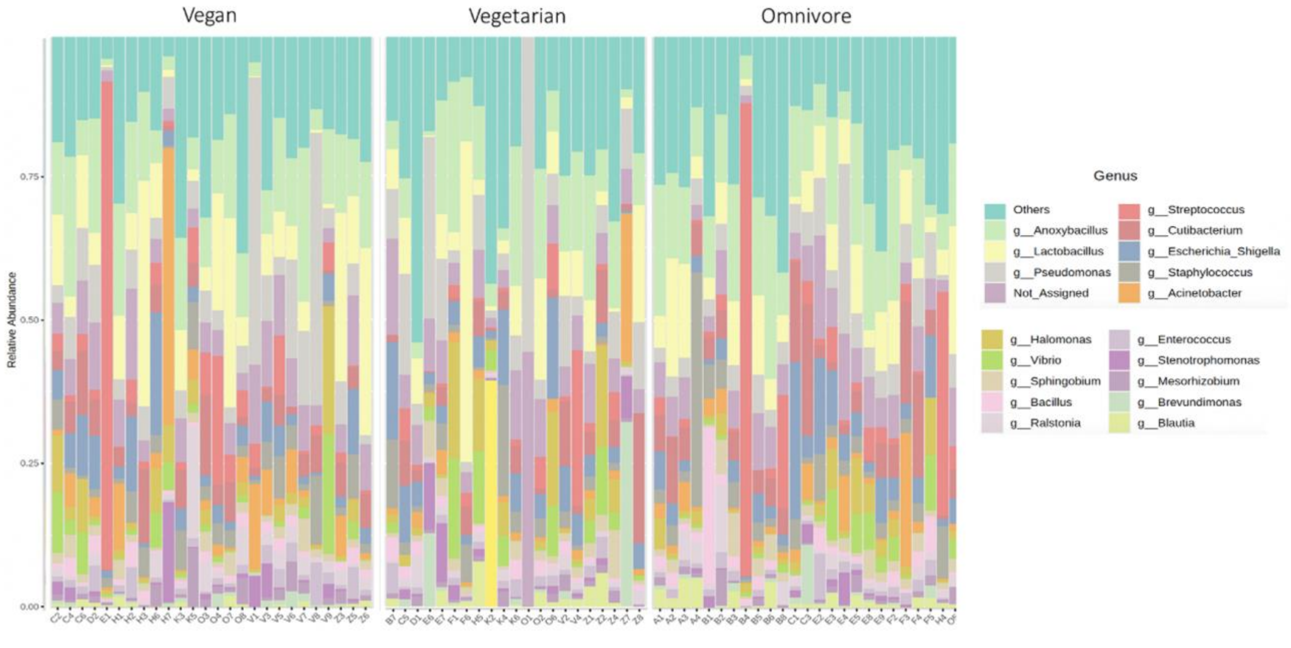

Table S1 Characteristics of the mothers and infants included in each diet group, including means and standard deviations.

Subject ID #	Participant Age(years)	Height(inches)	Weight(lbs.)	BMI	Infant Age(weeks)	Diet Category
C2	40	65	118	19.6	40.5	Non-vegetarians
C4	42	63.5	120	21.3	16	Non-vegetarians
C6	41	70	153	22	75	Non-vegetarians
D2	32	66	135	21.8	14	Non-vegetarians
E1	28	64	121	20.8	7.5	Non-vegetarians
E5	32	61	119	22.5	48	Non-vegetarians
E6	31	67	135	21.1	17	Non-vegetarians
E7	34	66	133	21.5	64	Non-vegetarians
H1	38	64	112	19.2	41.5	Non-vegetarians
H2	28	62	156	28.5	24.5	Non-vegetarians
H3	27	64	120	20.6	5	Non-vegetarians
H6	33	67	145	22.7	98.5	Non-vegetarians
H7	28	67	168	26.3	25	Non-vegetarians
K3	29	64	128	22	30.5	Non-vegetarians
K5	34	66	178	28.7	83	Non-vegetarians
O3	32	62	125	22.9	90	Non-vegetarians
O4	35	67	130	20.4	67	Non-vegetarians
O7	31	67	160	25.1	19	Non-vegetarians
O8	29	70	157	22.5	45.5	Non-vegetarians
V3	34	62	112	20.5	11	Non-vegetarians
V4	34	65	150	25	20	Non-vegetarians
V5	32	68.5	150	22.5	6.5	Non-vegetarians
V6	30	63	113	20	23	Non-vegetarians
V7	39	70	175	25.1	13.53	Non-vegetarians
V8	31	63	127	22.5	11	Non-vegetarians
V9	34	65	115	19.1	67	Non-vegetarians
Z3	36	66	146	23.6	39	Non-vegetarians
Z4	36	66	195	31.5	33.5	Non-vegetarians
Z5	18	62	105	19.2	17	Non-vegetarians
Z6	31	61	145	27.4	104	Non-vegetarians
Z8	35	61	135	25.5	68.5	Non-vegetarians
**Average**	32.70967742	65	138.0967742	22.9483871	39.53322581	
**STD**	4.762307203	2.648899142	22.18160625	3.088351337	29.35609345	

Subject ID #	Participant Age(years)	Height(inches)	Weight(lbs.)	BMI	Infant Age(weeks)	Diet Category
B7	29	63	160	28.3	16.5	Vegetarians
C5	26	66	125	20.2	93	Vegetarians
D1	33	66	140	22.6	23.5	Vegetarians
E2	27	67.5	138	21.3	27	Vegetarians
F6	28	64	151	25.9	12	Vegetarians
H5	31	66	150	24.2	9	Vegetarians
K2	29	66	190	30.7	17	Vegetarians
K4	38	62	121	22.1	50	Vegetarians
K6	32	69	154	22.7	97	Vegetarians
O1	31	66	140	22.6	12	Vegetarians
O2	34	63	125	22.1	186	Vegetarians
O6	37	64	153	26.3	28	Vegetarians
O9	25	65.5	133	21.8	43	Vegetarians
V1	28	63	112	19.8	82	Vegetarians
V2	46	67	125	19.6	126.5	Vegetarians
Z1	37	69	152	22.4	82	Vegetarians
Z2	33	61	110	20.8	58	Vegetarians
Z7	29	62	125	22.9	14	Vegetarians
**Average**	31.83333333	65	139.1111111	23.12777778	54.25	
**STD**	5.193321503	2.370157602	19.72424274	2.960883325	48.52750888	

Subject ID #	Participant Age(years)	Height(inches)	Weight(lbs.)	BMI	Infant Age(weeks)	Diet Category
A1	42	65	145	24.1	3.5	Vegan
A2	26	66	152	24.5	20.5	Vegans
A3	33	64.5	134	23	20.5	Vegans
A4	32	67	177	27.7	34.7	Vegans
B1	32	67	192	30.1	9	Vegans
B2	26	67	132	20.7	15	Vegans
B3	32	70	195	28	24.5	Vegans
B4	33	67	179	28	10	Vegans
B5	30	67	180	28.2	24	Vegans
B6	38	63	185	32.8	24.5	Vegans
B8	30	65	154	25.6	48	Vegans
B9	37	64	169	29	47	Vegans
C1	25	67	249	39	14	Vegans
C3	31	62	119	21.8	34	Vegans
E3	28	65	170	28.3	21	Vegans
E4	32	64	153	26.3	10	Vegans
E8	32	65	127	21.1	38	Vegans
E9	35	67	125	19.6	93	Vegans
F1	27	68.5	148	22.2	18	Vegans
F2	30	68	144	21.9	24	Vegans
F3	20	67	120	18.8	70	Vegans
F4	27	68	184	28	11	Vegans
F5	28	62	151	27.6	12.5	Vegans
H4	35	64	153	26.3	30.5	Vegans
O5	37	64	153	26.3	31.5	Vegans
**Average**	31.12	65.76	159.6	25.956	27.548	
**STD**	4.772490615	2.041853733	29.30159268	4.461229278	20.17195082	

Table S2 List of genera that were statistically different between omnivores and vegans.

Omnivore vs Vegan

Species	Discovery?	P value	Mean rank of Column A	Mean rank of Column B	Mean rank diff.	Mann-Whitney U	q value	# Samples found in	# Reads
g__Vermiphilaceae	Yes	0.000003	32.8	18.2	14.6	130	0.000053	38	14077
g__Dietzia	Yes	0.000004	17.3	33.7	-16.4	107.5	0.000065	7	1095
g__Mycobacterium	Yes	0.00001	32.9	18.1	14.8	127.5	0.00013	40	8856
g__Rothia	Yes	0.00001	32.5	18.5	14	137.5	0.00013	41	38050
g__Prevotellaceae_NK3B31_group	Yes	0.00001	18.5	32.5	-14	137.5	0.00013	1	4
g__Symplocastrum_CPER-KK1	Yes	0.00001	18.5	32.5	-14	137.5	0.00013	1	1772
g__Promicromonospora	Yes	0.000028	18.68	32.32	-13.64	142	0.000337	1	2387
g__Geobacillus	Yes	0.000029	32	19	13	150	0.000337	33	7267
f__Acidobacteriaceae_(Subgroup_1);__	Yes	0.000043	18.54	32.46	-13.92	138.5	0.000464	2	99
g__Actinomyces	Yes	0.000086	31.5	19.5	12	162.5	0.00085	35	9677
g__Brevibacillus	Yes	0.000086	31.5	19.5	12	162.5	0.00085	34	9613
d__Bacteria;__;__;__;__;__	Yes	0.000239	30.72	20.28	10.44	182	0.002169	44	2647
g__Muribaculum	Yes	0.000239	20.28	30.72	-10.44	182	0.002169	2	43
g__Halobacillus	Yes	0.000636	20.5	30.5	-10	187.5	0.00534	1	38
g__Candidatus_Stoquefichus	Yes	0.000636	20.5	30.5	-10	187.5	0.00534	1	20
g__Prevotella	Yes	0.000859	30.44	20.56	9.88	189	0.006938	36	8505
g__Dermabacter	Yes	0.001034	20.84	30.16	-9.32	196	0.008058	2	77
g__Bilophila	Yes	0.00131	20.56	30.44	-9.88	189	0.009852	3	295

Table S3 Beta diversity values for the PICRUSt functional analyses of the high and low groups for each of the fats (SF, UF, and TF).

	Bray Curtis PAIRWISE PERMANOVA			
	**KO**	**pseudo-F**	**p-value**	**q-value**
**KO**	Saturated Fat	1.23084	0.049	0.049
	Trans-Unsaturated Fat	1.36382	0.016	0.016
	Unsaturated Fat	1.05728	0.201	0.201
**EC**	Saturated Fat	1.13398	0.142	0.142
	Trans-Unsaturated Fat	1.54913	0.02	0.02
	Unsaturated Fat	0.91767	0.633	0.633
**Pathway**	Saturated Fat	1.40768	0.061	0.061
	Trans-Unsaturated Fat	1.89511	0.016	0.016
	Unsaturated Fat	1.12707	0.225	0.225

Table S4 Beta diversity values for the PICRUSt functional analyses of the diet groups (Vegan, Vegetarian, and Omnivore)

	Bray Curtis PAIRWISE PERMANOVA						
	**Group 1**	**Group 2**	**Sample size**	**Permutations**	**pseudo-F**	**p-value**	**q-value**
EC	Omnivore	Vegetarian	46	999	1.297615	0.052	0.156
		Vegan	51	999	0.906845	0.725	0.725
	Vegetarian	Vegan	47	999	0.929896	0.608	0.725
Pathway	Omnivore	Vegetarian	46	999	1.21249	0.167	0.501
		Vegan	51	999	0.931043	0.51	0.694
	Vegetarian	Vegan	47	999	0.814264	0.694	0.694
KO	Omnivore	Vegetarian	46	999	1.148471	0.079	0.237
		Vegan	51	999	0.934838	0.733	0.733
	Vegetarian	Vegan	47	999	0.92456	0.73	0.733

Table S5 Weighted NSTI values for EC and KO analysis for each sample.

Sample	EC	KO	Sample	EC	KO
A1	0.056106073	0.056106073	H2	0.03786123	0.03786123
A2	0.045283309	0.045283309	H3	0.03275183	0.03275183
A3	0.046098697	0.046098697	H4	0.02113892	0.02113892
A4	0.036470426	0.036470426	H5	0.04764815	0.04764815
B1	0.040096215	0.040096215	H6	0.02806144	0.02806144
B2	0.034495198	0.034495198	H7	0.03234479	0.03234479
B3	0.044653948	0.044653948	K2	0.12012476	0.12012476
B4	0.01886361	0.01886361	K3	0.06788261	0.06788261
B5	0.044103984	0.044103984	K4	0.03486434	0.03486434
B6	0.053097137	0.053097137	K5	0.03069208	0.03069208
B7	0.03282377	0.03282377	K6	0.04510838	0.04510838
B8	0.037959539	0.037959539	O1	0.01823645	0.01823645
C1	0.034278502	0.034278502	O2	0.05000208	0.05000208
C2	0.041946748	0.041946748	O3	0.03175927	0.03175927
C3	0.031450285	0.031450285	O4	0.03703039	0.03703039
C4	0.04854751	0.04854751	O5	0.03595756	0.03595756
C5	0.043630615	0.043630615	O6	0.04513616	0.04513616
C6	0.043016268	0.043016268	O7	0.032147	0.032147
D1	0.073918448	0.073918448	O8	0.07154066	0.07154066
D2	0.032936157	0.032936157	V1	0.02199263	0.02199263
E1	0.012942636	0.012942636	V2	0.05017757	0.05017757
E2	0.031500057	0.031500057	V3	0.0242485	0.0242485
E3	0.042885376	0.042885376	V4	0.05613388	0.05613388
E4	0.02886206	0.02886206	V5	0.04159648	0.04159648
E5	0.049261745	0.049261745	V6	0.03190081	0.03190081
E6	0.012350127	0.012350127	V7	0.04424171	0.04424171
E7	0.035953269	0.035953269	V8	0.03176458	0.03176458
E8	0.049654162	0.049654162	V9	0.06110107	0.06110107
E9	0.041627581	0.041627581	Z1	0.05283348	0.05283348
F1	0.053623363	0.053623363	Z2	0.05634457	0.05634457
F2	0.047910821	0.047910821	Z3	0.04070752	0.04070752
F3	0.047199775	0.047199775	Z4	0.03960766	0.03960766
F4	0.050540261	0.050540261	Z5	0.04133385	0.04133385
F5	0.054118803	0.054118803	Z6	0.04132942	0.04132942
F6	0.021502012	0.021502012	Z7	0.01626982	0.01626982
H1	0.055109122	0.055109122	Z8	0.07525042	0.07525042
